# The association between *ANKH* promoter polymorphism and chondrocalcinosis is independent of age and osteoarthritis: results of a case–control study

**DOI:** 10.1186/ar4453

**Published:** 2014-01-27

**Authors:** Abhishek Abhishek, Sally Doherty, Rose Maciewicz, Kenneth Muir, Weiya Zhang, Michael Doherty, Anna M Valdes

**Affiliations:** 1 Department of Rheumatology, Addenbrooke’s Hospital, Cambridge CB2 0QQ, UK; 2 Academic Rheumatology, University of Nottingham, Nottingham NG5 1PB, UK; 3 Respiratory and Inflammation iMed, AstraZeneca, MöIndal SE-431 83, Sweden; 4 Health Sciences Research Institute, University of Warwick, Warwick CV4 7AL, UK

## Abstract

**Introduction:**

Chondrocalcinosis (CC) most commonly results from calcium pyrophosphate crystal deposition (CPPD). The objective of this study is to examine the association between candidate single-nucleotide polymorphisms (SNPs) and radiographic CC.

**Methods:**

SNPs in ankylosis human (*ANKH)*, high ferritin (*HFE)*, tissue non-specific alkaline phosphatase (*TNAP)*, ecto-neucleotide pyrophosphatase 1 (*ENPP1),* and transferrin *(TE)* genes were genotyped in participants of the Genetics of Osteoarthritis and Lifestyle (GOAL) and Nottingham Osteoarthritis Case-Control studies. Adjusted genotype odds ratio (aOR_GENOTYPE_), the OR for association between one additional minor allele and CC, was calculated and adjusted for age, gender, body mass index (BMI), and osteoarthritis (OA) by using binary logistic regression. Statistical significance was set at *P* ≤0.003 after Bonferroni correction for multiple tests.

**Results:**

The -4bpG > A polymorphism in the 5′ untranslated region (5′ UTR) of *ANKH* associated with CC after Bonferroni correction. This was independent of age, gender, OA, and BMI; aOR_GENOTYPE_ (95% confidence interval, or CI) was 1.39 (1.14-1.69) (*P =* 0.001). rs3045 and rs875525, two other SNPs in *ANKH*, associated with CC; aOR_GENOTYPE_ (95% CI) values were 1.31 (1.09-1.58) (*P =* 0.005) and 1.18 (1.03-1.35) (*P* = 0.015), respectively; however, this was non-significant after Bonferroni correction.

**Conclusions:**

This study validates the association between a functional polymorphism in the 5′ UTR of *ANKH* and CC and shows for the first time that this is independent of age and OA – the two key risk factors for CC. It shows that other SNPs in *ANKH* may also associate with CC. This supports the role of extracellular inorganic pyrophosphate in the pathogenesis of CC. The findings of this hospital-based study require replication in a community-based population.

## Introduction

Chondrocalcinosis (CC) most commonly results from calcium pyrophosphate (CPP) crystal deposition (CPPD) [1]. CPPD may present as acute CCP crystal arthritis, CPPD with osteoarthritis (OA), chronic CPP crystal inflammatory arthritis, or asymptomatic CC [1]. Age, OA, diuretic use, and joint injury are recognized risk factors for CC [1,2]. Additionally, metabolic diseases that elevate extracellular pyrophosphate (ePPi) levels (hyperparathyroidism, hypomagnesemia, and hypophosphatasia), hemochromatosis, and familial predisposition are uncommon risk factors [1,2]. Though rare, familial predisposition is reported from several countries and different ethnic groups [3-9]. The pattern of inheritance is usually autosomal dominant. The main clinical phenotype is characterized by early onset (in the 20s or 30s) of acute CPP crystal arthritis with florid polyarticular CC and variable severity of accompanying structural arthritis/OA. However, a second phenotype with later onset in the sixth to seventh decades and oligo-articular CC that more closely resembles sporadic CPPD has also been reported [10]. This latter familial form may be more common than is recognized, the late onset of disease expression and geographic dispersal of families tending to mask such predisposition. An association with benign childhood fits appears unique to one UK family with early-onset polyarticular CC, and the responsible gene—CC gene 2 (*CCAL2*) on chromosome 5p15—was first identified in this family [11]. Other kindreds with CC due to mutations at this locus [3] have been reported, and the responsible gene was subsequently identified as the *ankylosis human (ANKH)* gene [12]. The other reported locus in an American family with premature OA and associated CPPD (CCAL1) is on chromosome 8q [13], and a specific gene predisposing to CPPD at this site has not been identified.

**Figure 1:**
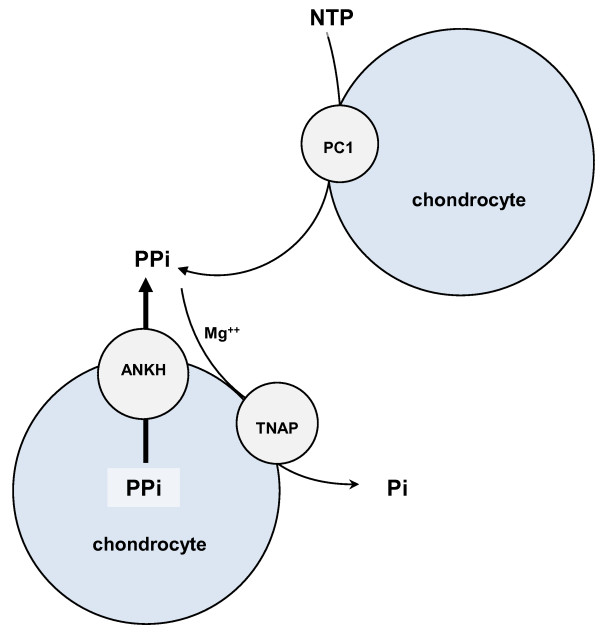
**Extracellular pyrophosphate metabolism.** ANKH, (progressive) ankylosis human; Mg^++^, magnesium; NTP, nucleotide tri-phosphate; PC1, plasma cell glycoprotein 1; Pi, phosphate; PPi, pyrophosphate; TNAP, tissue non-specific alkaline phosphatase.

Although several kindreds with familial CPPD are reported, the evidence for genetic contribution to sporadic CC is conflicting [3]. For example, the -4bpG > A transition in the 5′ untranslated region (5′ UTR) of *ANKH* (which encodes the trans-membrane PPi transport protein ANKH) associated with CC in a study of 128 CC cases and 475 healthy controls [14], whereas hereditary contribution to CC was not detected in a larger sibling study (n = 1,841) [15]. Therefore, the objectives of this study were to validate the reported association between -4bpG > A transition in the 5′ UTR of *ANKH* and CC, to examine whether this is independent of other risk factors of CC such as age and OA, and to investigate whether other candidate single-nucleotide polymorphisms (SNPs) in genes involved in PPi metabolism—*ANKH, tissue non-specific alkaline phosphatase (TNAP), and ectonucleotide pyrophosphatase 1 (ENPP1) (*Figure 1)—or iron overload (for example, *high ferritin and transferrin*) associate with CC.

## Methods

### Study design and participants

The case-control study used data from the Genetics of Osteoarthritis and Lifestyle (GOAL) and Nottingham Osteoarthritis Case-Control (NOAC) studies [16,17] (Table 1). The GOAL study was approved by the Nottinghamshire Research Ethics Committee (UK). The NOAC study was approved by the North Nottinghamshire Research Ethics Committee and the Leicestershire, Northamptonshire, and Rutland Research Ethics Committee. All study participants gave written informed consent. These studies, originally set up to examine the genetics of large-joint OA, recruited Caucasians with (a) hip or knee OA severe enough to require joint replacement surgery or (b) no radiographic or clinical features of hip or knee OA [16,17]. All participants were residents of Nottinghamshire, UK. Information about disease and demographic variables were collected at the study visit. Genomic DNA was extracted from the peripheral blood leukocytes by using standard protocols. GOAL study participants had radiographs of hands, knees, and pelvis; and the NOAC study participants had radiographs of the index knee or hip joint awaiting replacement. These radiographs were scored for CC at the knees, hips, wrists, and symphysis pubis and for metacarpophalangeal joint (MCPJ) calcification by a single trained observer (Sally Doherty).

**Table 1:** Demographics of study participants

	**CC present**	**CC absent**	
	**n = 658**	**n = 4,283**	***P***
Age in years, mean (SD)	70.89 (8.09)	67.58 (8.58)	<0.001
Female gender, number (percentage)	325 (49.4%)	2,240 (52.3%)	0.165
Body mass index in kg/m^2^, mean (SD)	28.52 (4.83)	29.48 (5.47)	<0.001
Knee or hip OA, number (percentage)	584 (90.0%)	3,378 (79.0%)	<0.001

CC, chondrocalcinosis; OA, osteoarthritis; SD, standard deviation.

### Cases and controls

Cases were participants with CC at any joint, whereas controls did not have CC at any joint x-rayed.

### Single-nucleotide polymorphism selection

Seventeen SNPs were selected on the basis of published associations with various phenotypes (Table 2).

**Table 2:** Association between all single-nucleotide polymorphisms and chondrocalcinosis in the Genetics of Osteoarthritis and Lifestyle study

	**SNP**	**Reason for selection (association with):**	**Minor allele frequency, %**		**OR**_**GENOTYPE **_**(95% CI)**	***P***
			**CC +**	**CC -**		
ANKH	-4bpG > A 5′ UTR	Chondrocalcinosis [14]	11.0	8.2	1.36 (1.07-1.73)	0.013
	rs3045	Rotator cuff tear and low intracellular PPi [18]	12.7	10.1	1.28 (1.02-1.60)	0.030
	rs39968	Parathyroid hormone level [19]	28.1	30.9	0.89 (0.76-1.05)	0.160
	rs875525	Parathyroid hormone level [19]	28.1	25.6	0.89 (0.76-1.04)	0.152
HFE	rs1800562	Hemochromatosis [20,21]	7.8	7.8	1.01 (0.77-1.33)	0.937
	rs1799945	Hemochromatosis [20,21]	17.1	14.7	1.20 (0.99-1.46)	0.070
TNAP	rs3200254	Increased TNAP activity [22]	11.1	11.0	0.99 (0.79-1.25)	0.957
	rs4654760	Rotator cuff tear [18]	6.7	6.6	1.01 (0.76-1.35)	0.955
ENPP1	rs1044498	Osteoarthritis [23]	13.7	12.5	1.07 (0.86-1.32)	0.564
	rs28933977	Osteoarthritis [23]	3.7	4.1	0.81 (0.55-1.20)	0.826
	rs1800949	Osteoarthritis [23]	25.7	26.2	0.98 (0.83-1.16)	0.298
	rs858342	Osteoarthritis [23]	24.1	25.1	0.92 (0.78-1.10)	0.365
	rs943003	Obesity [24]	40.6	41.9	1.06 (0.86-1.32)	0.401
Transferrin	rs1799852	Iron overload [25]	8.2	9.4	0.89 (0.69-1.15)	0.372
	rs2280673	Iron overload [25]	34.7	35.4	0.97 (0.83-1.12)	0.664
	rs3811647	Iron overload [25]	36.5	34.7	1.08 (0.92-1.26)	0.348

5′ UTR, 5′ untranslated region; ANKH, ankylosis human; CC, chondrocalcinosis; CI, confidence interval; ENPP1, ecto-neucleotide pyrophosphatase 1; HFE, high ferritin; OR_GENOTYPE_, genotype odds ratio; PPi, pyrophosphate; SNP, single-nucleotide polymorphism; TNAP, tissue non-specific alkaline phosphatase.

### Genotyping

Genotyping was carried out at AstraZeneca laboratories in Macclesfield, UK, by using the Taqman method and at Kbioscience Ltd (Hertfordshire, UK) by using the Kompetitive Allele Specific PCR (KASPar) chemistry. All 17 SNPs were genotyped in the GOAL study. All selected SNPs in any gene which contained at least one SNP that associated with CC with an uncorrected *P* ≤0.10 in the GOAL study were genotyped in the NOAC study participants.

### Covariates

Data about age (years), height (centimeters), and weight (kilograms) were collected at the study visit. Height and weight were used to calculate body mass index (BMI) (kg/m^2^). OA was defined as knee or hip OA clinically severe enough to warrant consideration of joint replacement surgery.

### Statistical analysis

Mean and standard deviation (SD) and number (percentage) were used for descriptive purposes. Chi-square test and student *t* test were used to compare categorical and continuous variables. Cases with CC were compared with controls without CC. All SNPs were checked for Hardy-Weinberg equilibrium (HWE). Data from the GOAL and NOAC studies were pooled together for analyzing genetic risk. Genotype odds ratio (OR_GENOTYPE_)—the OR for association between increasing number of minor alleles of an SNP and CC—was calculated. Binary logistic regression was used to calculate aOR_GENOTYPE_ (95% confidence interval, or CI) adjusting for age (tertiles), gender, BMI (tertiles), and OA. Additionally, OR_GENOTYPE_ was meta-analyzed with published studies by using fixed effects analysis given the lack of heterogeneity between studies. Meta-analyses were performed by using R V.2.13.1 [26]. Other analyses were carried out by using SPSSv14. Statistical significance for genetic association was set at *P* ≤0.003 after application of Bonferroni correction for multiple tests. Linkage disequilibrium for the four *ANKH* SNPs studied was estimated from unphased genotype data by using the Haploview 4.2 version [27].

## Results and discussion

The descriptive characteristics of study participants are presented in Table 1. Three thousand one hundred forty-one GOAL and 1,800 NOAC study participants were included. The mean age (SD), number of females (percentage), and mean BMI (SD) of the GOAL study and NOAC study participants were 66.6 (7.9) and 70.5 (9.2) years, 1,520 (48.4%) and 1,045 (58.1%) women, and 29.3 (5.3) and 29.5 (5.6) kg/m^2^, respectively. GOAL study participants were significantly younger (*P* <0.001), were less likely to be female (*P* <0.001), and had similar BMI (*P* = 0.22) compared with the NOAC study participants.

Six hundred fifty-eight (13.2%) participants had CC at any site. All genotyped SNPs were in HWE. As TNAP571 [28] was a rare mutation in the GOAL study population (minor allele frequency = 0.01), it was excluded from further analysis. Three *ANKH* and one *HFE* SNPs associated with CC with an uncorrected *P* ≤0.10 in the GOAL study population (Table 2). Thus, all *ANKH* (-4bpG > A 5′ UTR, rs3045, rs39968, and rs875525) and *HFE* (rs1800562 and rs1799945) SNPs genotyped in the GOAL study were further genotyped in the NOAC study participants. The four *ANKH* SNPs were not in linkage disequilibrium (Table 3). Among the six SNPs genotyped in both the GOAL and NOAC studies, the -4bpG > A 5′ UTR of *ANKH* associated with CC after adjustment for age, gender, BMI, and OA; aOR_GENOTYPE_ was 1.39 (1.14-1.69) (*P =* 0.001) (Table 4). There was a trend toward an association between rs3045 and CC; aOR_GENOTYPE_ was 1.31 (1.09-1.58) (*P =* 0.005); however, it was not statistically significant after application of Bonferroni correction (Table 4). The frequency of rare and common allelic variants in the ANKH and HFE SNPs genotyped in both the GOAL and NOAC studies is shown in an additional file (Additional file 1: Table S1).

**Table 3:** **Linkage disequilibrium between the genotyped single-nucleotide polymorphisms in ankylosis human ( *ANKH *) gene**

**Locus 1**	**Locus 2**	**D′**	**r**^**2**^
rs39968	rs3045	0.746	0.028
rs39968	rs875525	0.268	0.011
rs39968	-4bpG > A 5′ UTR	0.076	0.001
rs3045	rs875525	0.826	0.226
rs3045	-4bpG > A 5′ UTR	0.625	0.004
rs875525	-4bpG > A 5′ UTR	0.448	0.006

5′ UTR, 5′ untranslated region.

**Table 4:** Association between chondrocalcinosis and single-nucleotide polymorphisms in the combined dataset

	**Common allele**	**Rare allele**	**MAF, %**		**OR**_**GENOTYPE **_**(95% CI)**	***P***	**aOR**_**GENOTYPE **_**(95% CI)**	***P***
			**CC+**	**CC-**				
-4bpG > A 5′ UTR	G:G	A:A	10.6	8.1	1.36 (1.12-1.65)	0.002	1.39 (1.14-1.69)	0.001
rs3045	T:T	C:C	12.4	9.9	1.29 (1.08-1.55)	0.006	1.31 (1.09-1.58)	0.005
rs39968	C:C	T:T	28.8	30.8	0.91 (0.80-1.04)	0.155	0.91 (0.80-1.04)	0.156
rs875525	C:C	T:T	28.1	25.0	1.18 (1.03-1.34)	0.014	1.18 (1.03-1.35)	0.015
rs1800562	G:G	A:A	8.3	7.6	1.10 (0.89-1.36)	0.386	1.13 (0.91-1.40)	0.273
rs1799945	C:C	G:G	16.9	14.6	1.19 (1.02-1.39)	0.032	1.18 (1.00-1.38)	0.047

5′ UTR, 5′ untranslated region; aOR_GENOTYPE_, adjusted genotype odds ratio; CC, chondrocalcinosis; CI, confidence interval; MAF, minor allele frequency; OR_GENOTYPE_, genotype odds ratio.

### Comparison with published studies

Two studies have reported on the association between CC and *HFE* SNPs [20,21]. Of these, one study reported genotype data about rs1800562, and rs1799945 without reporting full data on compound heterozygote numbers [20], whereas the other did not report detailed genotype data [21]. Therefore, the latter study was not included in the meta-analysis. In a meta-analysis of the data of the present study and of the study by Alizadeh et al. [20], the values for pooled OR_GENOTYPE_ (95% CI) for association between rs1799945 and rs1800562 and CC were 1.20 (1.04-1.39) (*P* = 0.015) and 1.08 (0.88-1.33) (*P =* 0.445), respectively. Similarly, the results of the present study and the previous study that reported an association between CC and -4bpG > A transition in the 5′ UTR of ANKH were meta-analyzed [14]. The pooled OR_GENOTYPE_ (95% CI) was 1.36 (1.13-1.61) (*P =* 0.001).

The present study confirms the association between CC and -4bpG > A transition in the 5′ UTR of *ANKH.* It is the first study to show that this association is independent of age and OA, which are the two major established risk factors of CC. This study also raises the possibility that other SNPs in ANKH (for example, rs3045) may also associate with CC. However, in keeping with previous reports, there was no association between SNPs in *TNAP* or *ENPP1* and CC [29].

*ANKH* encodes a multipass transmembrane protein (ANKH) in joints and other tissues and participates in the export of intracellular PPi [30,31]. PPi cannot diffuse across cell membranes passively, and ANKH is the principal way in which intracellular PPi reaches the extracellular environment. ANKH-mediated control of PPi levels regulates tissue calcification and susceptibility to arthritis [30,31]. The autosomal dominant mutations in *ANKH* are thought to confer a gain in PPi transport function leading to increased extracellular PPi levels [14,32]. Functional assays show that the -4bpG > A transition in the 5′ UTR of *ANKH* reduces intracellular PPi (a surrogate for increased transcellular PPi export) and increases ANKH expression *in vitro*[14,18]. The minor alleles of rs3045 also result in lower intracellular PPi levels *in vitro*, providing external validity to our finding of a possible association between this polymorphism and CC [18]. These two SNPs are not in linkage disequilibrium. The minor allele frequency for the -4bpG > A transition in 5′ UTR of *ANKH* and rs3045 is higher in this study than that in the multi-ethnic 1000 Genomes Project. (See Additional file 2: Table S2 for genotype frequencies of the selected SNPs in the 1000 Genomes Project). This may explain, in part, why CPPD is more common in Caucasians than in other ethnicities.

Though not statistically significant, results from the data suggest that rs1799945 (*HFE* SNP associated with smaller iron overload), but not rs1800562 (*HFE* SNP associated with greater iron overload), may associate with CC. Similar findings have been reported previously [20]. The lack of association between homozygosity for minor allele at rs1800562 and CC may be due to a channeling bias (that is, patients with hemochromatosis are excluded from these studies).

This is the largest reported study of genetic risk factors for CC. This study has several strengths. First, the analysis of genetic risk was adjusted for factors that associate with CC, specifically age and OA, and also for gender, which associates with iron overload. Moreover, correction for multiple testing was applied to reduce the chances of a type I error. However, this study has several caveats. First, this is a hospital-based study carried out by reconstituting cases and controls within cohorts assembled primarily to examine risk factors for knee or hip OA within the East Midlands region of the UK. Cases with mild to moderate large-joint OA were not included. The study sample therefore does not resemble a community-based population and is restricted to one area of the country. Moreover, as more than 78% of participants had severe large-joint OA, the results may be confounded by their OA status. However, to minimize any confounding and to improve the generalizability of these findings, we have adjusted for OA at the knee and hip. Second, participants of the NOAC study did not have the same extensive radiographic phenotyping of CC as participants of the GOAL study. As a result, some NOAC study participants who may have CC at distant joints without CC at the joint to be replaced will be misclassified as ‘CC negative’ controls. This misclassification is likely to minimize the genetic association and does not invalidate the genetic associations observed in this study.

## Conclusions

This study validates an established genetic association with CC and shows that this is independent of age and OA. This study also raises the possibility that other SNPs in *ANKH* associate with CC. Larger studies with greater power are required to confirm these findings. Finally, the findings of this study derived from a hospital-based cohort warrant confirmation in a community-based population including cases with mild to moderate disease and in other countries.

## Abbreviations

5′ UTR: 5′ untranslated region; ANKH: ankylosis human; BMI: body mass index; CC: chondrocalcinosis; CCAL (1 or 2): chondrocalcinosis (1 or 2) gene; CI: confidence interval; CPP: calcium pyrophosphate crystal; CPPD: calcium pyrophosphate crystal deposition; ENPP1: ectonucleotide pyrophosphatase 1; GOAL: Genetics of Osteoarthritis and Lifestyle; HFE: high ferritin; HWE: Hardy-Weinberg equilibrium; MCPJ: metacarpophalangeal joint; NOAC: Nottingham Osteoarthritis Case-Control; OA: osteoarthritis; ORGENOTYPE: genotype odds ratio; PPi: pyrophosphate; SD: standard deviation; SNP: single-nucleotide polymorphism; TNAP: tissue non-specific alkaline phosphatase.

## Competing interests

RM is an employee of AstraZeneca (London, UK), owns shares in that company, and was involved as a scientific collaborator in the GOAL study. She is named on a patent application for a gene associated with osteoarthritis. The other authors declare that they have no competing interests. AstraZeneca had no role in the study design, data analysis, or drafting of the manuscript.

## Authors’ contributions

AA, RM, and WZ were involved in study conception and design and in analysis and interpretation of the data. KM and MD were involved in study conception and design and in acquisition, analysis, and interpretation of the data. AMV was involved in study conception and design and in acquisition, analysis, and interpretation of the data and carried out the meta-analysis. SD was involved in study conception, data acquisition, and radiographic scoring. All authors were involved in drafting the article or revising it for important intellectual content. All authors read and approved the final version of the manuscript.

## Authors’ information

AMV and MD are joint senior authors.

## Supplementary Material

Additional file 1: Table S1 Genotypes frequencies of ankylosis human (*ANKH*) and high ferritin (*HFE*) single-nucleotide polymorphisms (SNPs) in Genetics of Osteoarthritis and Lifestyle (GOAL) and Nottingham Osteoarthritis Case-Control (NOAC) studies. Click here for file

Additional file 2: Table S2 Published minor allele frequency (MAF) of selected single-nucleotide polymorphisms (SNPs) from the 1000 Genomes Project phase 1. Genotype data from 1,094 individuals from across the world (http://www.1000genomes.org/node/506). Click here for file
