# Supplementary material for: The association between ANKH promoter polymorphism and chondrocalcinosis is independent of age and osteoarthritis: results of a case–control study
Source: Arthritis Res Ther. 2014 Jan 27;16(1):R25. doi: 10.1186/ar4453 (PMC3978851; doi:10.1186/ar4453)
Supplement: Additional file 1: Table S1 — Genotypes frequencies of ankylosis human (ANKH) and high ferritin (HFE) single-nucleotide polymorphisms (SNPs) in Genetics of Osteoarthritis and Lifestyle (GOAL) and Nottingham Osteoarthritis Case-Control (NOAC) studies. [file ar4453-S1.doc]

Supplementary Table 1: Genotypes frequencies of *ANKH*, and *HFE* SNPs in GOAL and NOAC studies

| SNP | Chondrocalcinosis + | | |  | Chondrocalcinosis - | | |
| --- | --- | --- | --- | --- | --- | --- | --- |
|  | 1:1 | 1:2 | 2:2 |  | 1:1 | 1:2 | 2:2 |
| -4bpG>A 5’UTR | 517 | 124 | 7 |  | 3554 | 628 | 26 |
| rs3045 | 495 | 149 | 6 |  | 3422 | 764 | 37 |
| rs39968 | 324 | 281 | 48 |  | 2008 | 1842 | 384 |
| rs875525 | 336 | 265 | 51 |  | 2365 | 1587 | 258 |
| rs1800562 | 548 | 91 | 8 |  | 3598 | 588 | 25 |
| rs1799945 | 446 | 182 | 18 |  | 3059 | 1041 | 91 |
