# Supplementary material for: The association between ANKH promoter polymorphism and chondrocalcinosis is independent of age and osteoarthritis: results of a case–control study
Source: Arthritis Res Ther. 2014 Jan 27;16(1):R25. doi: 10.1186/ar4453 (PMC3978851; doi:10.1186/ar4453)
Supplement: Additional file 2: Table S2 — Published minor allele frequency (MAF) of selected single-nucleotide polymorphisms (SNPs) from the 1000 Genomes Project phase 1. Genotype data from 1,094 individuals from across the world (http://www.1000genomes.org/node/506). [file ar4453-S2.doc]

**Supplementary table 2:** Published minor allele frequency (MAF) of selected SNPs from the 1000 genomes project phase 1 genotype data from 1094 worldwide individuals (<http://www.1000genomes.org/node/506>)

| Gene | SNP | MAF (%) |
| --- | --- | --- |
| ANKH | rs139418249^1^ | 0.045 |
|  | rs3045 | 0.056 |
|  | rs39968 | 0.331 |
|  | rs875525 | 0.336 |
| HFE | rs1800562 | 0.020 |
|  | rs1799945 | 0.084 |
| TNAP | rs3200254 | 0.279 |
|  | rs4654760 | 0.190 |
| ENPP1 | rs1044498 | 0.292 |
|  | rs28933977 | 0.024 |
|  | rs1800949 | 0.166 |
|  | rs858342 | 0.200 |
|  | rs943003 | 0.320 |
| Transferrin | rs1799852 | 0.140 |
|  | rs2280673 | 0.441 |
|  | rs3811647 | 0.339 |

^1^The MAF of rs139418249 (-4bp G>A in 5’ UTR) was higher at 7.9% in UK blood donors [15].
